# New identification of the moray eel *Gymnothorax minor* (Temminck & Schlegel, 1846) in China (Anguilliformes, Muraenidae)

**DOI:** 10.3897/zookeys.752.24231

**Published:** 2018-04-23

**Authors:** Yuan Li, Liyan Zhang, Linlin Zhao, Ji Feng, Karhoe Loh, Xinqing Zheng, Longshan Lin

**Affiliations:** 1 Third Institute of Oceanography, State Oceanic Administration, Xiamen, Fujian 361005, China; 2 Fujian Institute of Oceanography, Xiamen, Fujian 361013, China; 3 F; 4 irst Institute of Oceanography, State Oceanic Administration, Qingdao, Shandong 266003, China; 5 Institute of Ocean and Earth Sciences, University of Malaya, K; 6 uala Lumpur, Selangor 50603, Malaysia

**Keywords:** DNA barcoding; geographical distribution; *Gymnothorax reticularis*; morphological characteristics; species taxonomy

## Abstract

A new identification of *Gymnothorax minor* (Temminck & Schlegel, 1846) is documented based on morphological characteristics and DNA barcoding. Sixty-one individuals of *G. minor* were collected from the East China Sea and the South China Sea. This species was previously reported as *Gymnothorax reticularis* Bloch, 1795 in China because of the similarity in external shape and color. *Gymnothorax minor* can be easily distinguished from *G. reticularis* by its color pattern of 18–20 irregular dark brown vertical bars and the body having scattered small brown spots. Additionally, the teeth are uniserial on both jaws, and the vertebrae number 137–139. By combining congener sequences of the cytochrome oxidase I (COI) gene from GenBank, two groups were detected among all the COI sequences of the currently named *G. minor*, which further indicated that two valid species were present based on genetic distance. A divergence also occurred on the number of vertebrae between the northern and southern populations. The phylogenetic and morphological analysis strongly supports that the northern and southern populations of *G. minor* are two different species. Furthermore, the distribution area of the northern *G. minor* has expanded southward to 5°15'N in the South China Sea. More specimens of *G. minor* and *G. reticularis* are crucial in order to define their geographical distribution boundaries and provide the correct DNA barcoding.

## Introduction

Moray eels are distributed in the subtropical and tropical seas, which are not well studied because of their cryptic habitats and occasionally aggressive behaviors. The genus *Gymnothorax* is regarded as a polyphyletic assemblage of ungrouped moray eels and can be easily distinguished from homologous species with irregular vertical bars along the dorsal midline before the dorsal fin origin (Smith 2012). However, *Gymnothorax minor* (Temminck & Schlegel, 1846) has been often confused with *Gymnothorax reticularis* Bloch, 1795 because of the similar morphological characteristics. More regional taxonomic reviews of *G. minor* can be found from Japan (Tawa and Mochioka 2009, Yamada et al. 2009, Nakabo 2013), Korea (Kim et al. 2012), Vietnam (Hibino et al. 2016), the Philippines (Bucol and Alcala 2015, Wagey et al. 2015), Australia, and New Zealand (Böhlke and McCosker 2001). A total of 37 species of the genus *Gymnothorax* exist, but *G. minor* has been reported rarely in China (Chen and Zhang 2015), while *G. reticularis* is the widely used identification by Chinese ichthyologist. The Chinese name “Wang Wen Luo Xiong Shan” was assigned to the species “*Gymnothorax reticularis*”, which has been persistently confused with *G. minor* (Zhu et al. 1962, 1985, Cheng and Zheng 1987, Zhang et al. 2010, Chen and Zhang 2015). In fact, both species can be easily distinguished by the number of vertebrae and their differing distribution ranges: the vertebrae number 129–143 in *G. minor* vs. 114–126 in *G. reticularis*; *G. minor* is found from the northwestern to the southwestern Pacific vs. *G. reticularis* from the Indian Ocean to the Red Sea (Smith and Böhlke 1997, Kim et al. 2012). Therefore, the identification of these species in Chinese waters must be clarified based on actual specimens. During our ichthyofaunal surveys, we initially identified the moray eels as “*Gymnothorax reticularis*” by mistakes in 2012 and 2013. With our further research, we were fortunate to find this wrong identification and correct it. By now we have 61 individuals of *G. minor* found from the East China Sea and the South China Sea.

DNA barcoding, the mtDNA gene cytochrome c oxidase subunit I (COI) used in molecular taxonomy can help expand our knowledge by discriminating among species (Domingues et al. 2013, Puckridge et al. 2013), discovering newly recorded and new species (Xiao et al. 2016), revealing cryptic species (Hajibabaei et al. 2007, Zemlak et al. 2009), and identifying ichthyoplankton (Ko et al. 2013, Hubert et al. 2015, Li et al. 2017), which can also be sequenced with universal primers (Hebert et al. 2003). In the present study, DNA barcoding was employed to better solve the taxonomic problems of *Gymnothorax* at species level. Not surprisingly, misidentified DNA barcoding of these species has been found in GenBank, calling for correct identifications.

One objective of this study is to report the species of *G. minor* as the new identification with its new distribution in China; the other is to describe this species based on morphological characteristics and DNA barcoding, and to correct the current COI sequences of this species released in GenBank. The results will highlight the need for caution when identifying moray eels and will facilitate the fishery management, biodiversity conservation, and sustainable exploitation of this species.

## Materials and methods

### Specimen collection

**Figure 1.**
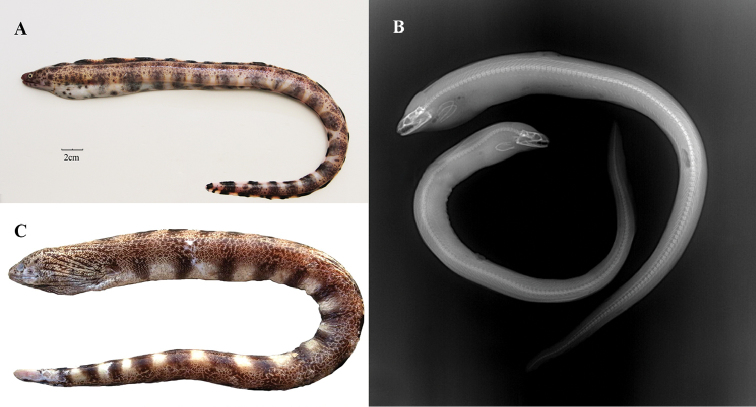
*Gymnothorax minor* (**A**) and X-ray photo (**B**), *Gymnothorax reticularis* (**C**). Photograph of *G. reticularis* is from Stern and Goren (2013).

A total of 61 individuals of *G. minor* was collected from the East China Sea and the South China Sea from September 2012 to November 2017 (Figure 1, Table 1). All specimens were identified based on morphological characteristics as defined by Nakabo (2013) and Yamada et al. (2009). For genetic studies, a piece of muscle tissue was obtained from randomly selected individuals and preserved in 95% ethanol. All examined specimens were preserved at the Third Institute of Oceanography, State Oceanic Administration.

**Table 1.** Information on the moray eel specimens and sequences in this study.

Species	This study						Cited accession no.
	Sea	Number	Latitude (N) / Longitude (E)	Range of total length (mm)	Range of weight (g)	Accession no.	
*Gymnothorax minor*	South China Sea (2012)	12	18°, 110°	347.4–481.9	42.1–180.2	MG755739-MG755740	HQ122466, KF681855
		1	17°30', 110°	396.9	74.3		
		1	5°15', 110°	362.2	190.2		
	South China Sea (2013)	3	18°, 109°	266.3–299.6	81.1–134.0		
		5	17°30', 109°	306.9–495.6	46.2–195.1		
		1	17°, 109°	404.3	154.2		
		23	18°, 110°	300.9–526.5	48.5–196.5		
		2	8°30', 109°	332.3–432.0	71.6–94.4		
		1	5°30', 109°30'	447.8	145.1		
		7	6°, 109°	375.2–453.1	94.3–142.8		
	East China Sea (2017)	5	24°26', 118°05'	406.2–552.5	97.3–180.1	MG755735-MG755738, MG755744	
*G. reticularis*	–						HM461876, KU942701, KU942736, KU942739, KU942760-KU942762, KX215183, KX215184, MG220570
*G. buroensis*	–						JQ350022, JQ431789, KF929925
*G. reevesii*	–						EF607396, EU595145, FJ237992
*G. fimbriatus*	–						KF929928
*Muraenesox cinereus*	–						HM068292, KU942795, KX215196
*Uropterygius fuscoguttatus*	–						HQ122477, JQ350410, JQ432206

### Morphological analysis

Counting and measurement methods were performed as described by Böhlke (1989). The counts included the following characteristics: bars behind gill opening, teeth, dentition, median intermaxillary teeth, dorsal fin origin, and vertebrae (counted from X-ray photos). The measurements included the following traits: total length, pre-anal length, depth at gill-opening, depth at anus, width at gill-opening, width at anus, head length, snout length, eye diameter, and interorbital width. All measurements were performed to the nearest 0.1 mm using calipers. Color and brown brands/spots were documented in fresh fish, and all remaining measurements were implemented on preserved specimens.

### Molecular analyses

Five individuals were randomly chosen from each survey for genetic analysis. Genomic DNA was isolated from muscle tissue by proteinase K digestion and extracted with Qiagen DNeasy kit. The fragment of mitochondrial DNA COI was amplified using the primers F1: 5’-TCAACCAACCACAAAGACATTGGCAC-3’; and R1: 5’-TAGACTTCTGGGTGGCCAAAGAATCA-3’ (Ward et al. 2005). Each polymerase chain reaction (PCR) was performed in a 25 μL reaction mixture containing 17.5 μL of ultrapure water, 2.5 μL of 10×PCR buffer, 2 μL of dNTPs, 1 μL of each primer (5 μM), 0.15 μL of Taq polymerase, and 1 μL of DNA template. PCR amplification was performed in a Biometra thermal cycler under the following conditions: 5 min of initial denaturation at 95 °C; 30 cycles of 45 s at 94 °C for denaturation, 45 s at 52 °C for annealing, and 45 s at 72 °C for extension; and a final extension at 72 °C for 10 min. The PCR products were purified and sequenced by Personal Biotechnology Co., Ltd.

To determine the right DNA barcoding of *G. minor*, homologous COI sequences were downloaded from GenBank for comparative analysis (Table 1). The sequences were aligned using DNASTAR software (Madison, WI, USA). A neighbor-joining (NJ) tree was built and the distances between and within species were calculated using MEGA 5.0 (Tamura et al. 2011) with 1,000 bootstrapping replications under the best selected K2P model. The DNA barcoding gap was calculated for all species, which is the maximum intraspecific distance of each species against its minimum distance to the nearest neighbor (Ward et al. 2005). *Uropterygius fuscoguttatus* and *Muraenesox cinereus* were chosen as outgroup.

## Results

### Morphological analysis

Counts and measurements from 61 individuals of *G. minor* were conducted and the generally morphological characteristics of this species are presented in Figure 1 and Table 1. The total length ranged from 266.3 to 552.5 mm, and the range of weight was from 42.1 to 196.5 g. This species can be described as the following combination of characteristics referred to Kim et al. (2012):

Measurements presented as percentages of total length (%): head length 13.5–15.3, pre-anal length 45.1–52.1, depth at gill-opening 4.5–5.2, depth at anus 4.4–5.0, width at gill-opening 2.6–3.9, width at anus 3.4–3.8. Measurements presented as percentages of head length (%): snout length 13.4–15.2, eye diameter 9.2–10.2, interorbital width 12.1–14.8.

Body naked, elongate, slightly compressed, tapering toward the tail. Head with many wrinkles, mouth terminal, snout blunt and rounded. Gill opening a small slit. Nostrils two pairs, posterior nostrils small and oval, while anterior nostrils narrow and tubular. Cephalic pores minute; supraorbital pores three; four infraorbital pores along the upper jaw; six mandibular pores along the lower jaw; two branchial pores. Both sets of jaw teeth serrated. Mandibular teeth 14 in a single row, tapering in size posteriorly. One median intermaxillary tooth; vomerine teeth small blunt, 12 in a single row. Maxillary teeth 15 in a single row, tapering in size posteriorly. Dorsal fin origin slightly before the gill opening. Pectoral fin absent. Caudal fin small, confluent with dorsal and anal fins. Lateral line greatly reduced, pores inconspicuous. Anus located almost in the middle of the body. Vertebrae 137–139.

Head and body pale yellowish, head with dark markings, body with scattered small brown spots, 18–20 irregular dark brown vertical bars from behind the gill opening to the caudal fin margin, and the bars may be diffuse and often indistinct.

### Molecular analyses

The COI gene fragments of five *G. minor* individuals randomly chosen from each survey were sequenced and edited. All newly amplified sequences were submitted to GenBank with the accession numbers MG755735-MG755744. A set of homologous sequences were downloaded from GenBank and 36 sequences in total were used for analysis with 553 bp in length. Within all sequences, 196 variable sites, 187 parsimony-informative sites, and nine singleton sites were detected, and four deletions/insertions were observed. The A+T content (55.7%) was higher than that of G+C (44.3%), revealing a slight base against G+C.

**Figure 2.**
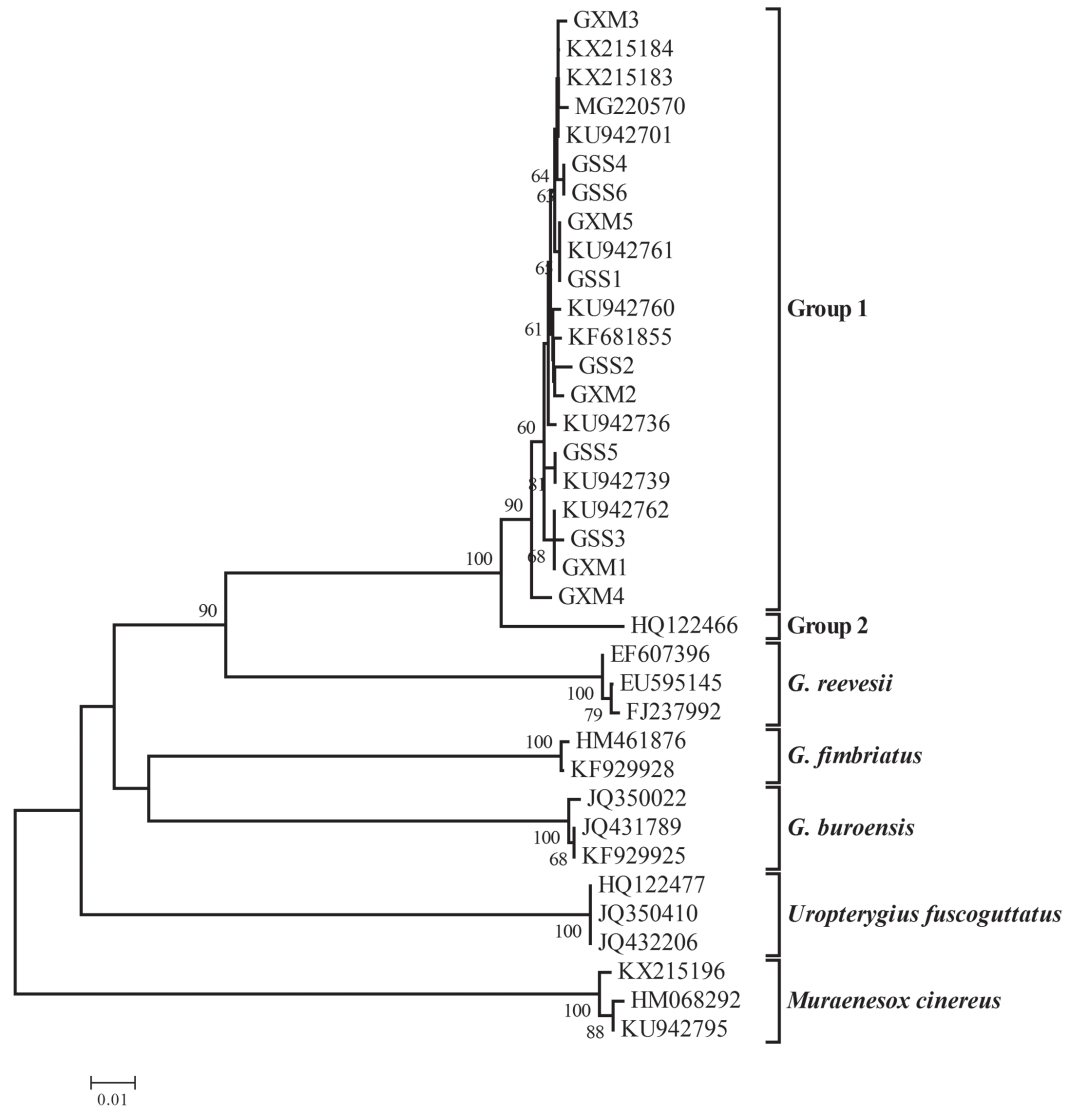
NJ tree of moray eels constructed with MEGA based on the K2P model. Bootstrap values of > 50% from 1,000 replications are shown.

An NJ tree of the *Gymnothorax* species group was constructed based on the K2P model (Figure 2). *U. fuscoguttatus* and *M. cinereus* were chosen as outgroups. The results showed that five groups were found in the NJ tree with high bootstrap values, supporting the existence of division. *Gymnothorax buroensis*, *G. reevesii*, and *G. fimbriatus* were clearly clustered together, while *G. minor* and *G. reticularis* were mixed together and formed two groups. Except for HM461876, the remaining *G. reticularis* sequences and KF681855 named *G. minor* clustered with our *G. minor* to form Group 1, suggesting that the specimens currently named *G. reticularis* should actually be identified as *G. minor*. HM461876 was submitted under the name “*Gymnothorax reticularis*”, but it clustered with *G. fimbriatus*. HQ122466 formed Group 2 and was identified as *G. minor* collected from Australian.

Based on the K2P model, the genetic distances of COI within and between groups were computed (Table 2). The mean genetic distance within each group ranged from 0 to 0.6%, while the genetic distance between Group 1 and Group 2 was 3.8%, exceeding the threshold of species delimitation (approximately 2%) (Hebert et al. 2003), suggesting they were different valid species. Therefore, these five groups in the NJ tree should be five different and valid species.

**Figure 3.**
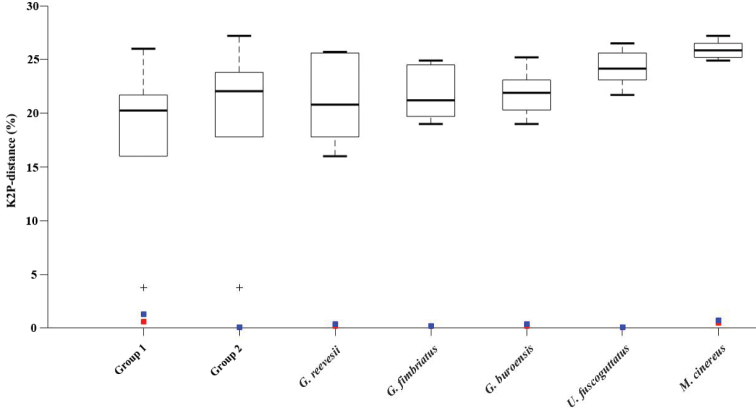
DNA barcoding gaps for all of the species based on the K2P model. Median interspecific distances with maximum and minimum values are represented by the upper and lower bars, respectively. Blue square: maximum intraspecific distance; Red square: mean intraspecific distance.

The maximum intraspecific distance of each species ranged from 0–1.3%, while the minimum interspecific distances of all species were higher than 2%. The species discrimination power of DNA barcoding was demonstrated by the barcoding gaps that were drawn for all species on the basis of the K2P distances shown in Figure 3. Because the latter value was always higher than the former one, overlaps were not detected in all species.

**Table 2.** Genetic distances of COI within (on the diagonal in bold) and between (below the diagonal) groups.

	Group 1	Group 2	*G. reevesii*	*G. fimbriatus*	*G. buroensis*	*U. fuscoguttatus*	*M. cinereus*
Group 1	**0.006**						
Group 2	0.038	–					
*G. reevesii*	0.160	0.178	**0.002**				
*G. fimbriatus*	0.202	0.222	0.197	**0.002**			
*G. buroensis*	0.203	0.219	0.219	0.190	**0.002**		
*U. fuscoguttatus*	0.217	0.238	0.256	0.245	0.231	**0**	
*M. cinereus*	0.260	0.272	0.257	0.249	0.252	0.265	**0.005**

## Discussion

The moray eels with irregular dark vertical bars on the body and serrated teeth on jaws and the intermaxillary region are defined as *Gymnothorax reticularis* species group. *Gymnothorax minor* and *G. reticularis* are common species in the assembled species group, and have usually been confused by many ichthyologists because of the similarity of external shape and color. However, the two species can be easily distinguished by their numbers of vertebrae and distribution range.

The moray eel collected from Chinese waters were initially identified as *G. reticularis* according to the descriptions of previous reports (Zhu et al. 1962, 1985, Cheng and Zheng 1987, Zhang et al. 2010, Chen and Zhang 2015), which included only simple external descriptions without vertebra number. The morphological analysis based on newly collected specimens in this study showed that the vertebra number ranged from 137 to 139, similar to that of *G. minor* (129–143) but obviously different from *G. reticularis* (114–126) (Smith and Böhlke 1997). Other morphological characteristics of *G. minor* collected from Chinese waters were highly consistent with the original morphological description (Temminck and Schlegel 1846) and the subsequent report of the lectotype of *G. minor* (Boeseman 1947). Considering the geographical distribution, the moray eel in Chinese waters was further shown to be *G. minor*.

By molecular analysis, it is shown that DNA barcoding is effective and reliable to identify the *Gymnothorax* species. Furthermore, a ten-fold sequence divergence between the average interspecific and the average intraspecific difference was detected because of the existence of barcoding gap, and this divergence has been suggested to be the standard COI threshold for species identification (Hebert et al. 2003). From the NJ tree, all COI sequences of *G. minor* in GenBank are now correctly identified; HM461876 as *G. reticularis* is in fact *G. fimbriatus*, while the other COI sequences of *G. reticularis* are clearly attributable to *G. minor*. The specimen (HQ122466) is acquired from Australian Museum (specimen voucher 35980) and identified as *G. minor*. Considering the collected geographical location, we can confirm the moray eel is unquestionably *G. minor* instead of *G. reticularis*. Unfortunately, the correct COI sequences of *G. reticularis* is absent in GenBank and the genetic relationship between *G. minor* and *G. reticularis* has not been evaluated.

The moray eel *G. minor* has been known as an anti-tropical species and initial geographical distribution was reported in northwestern Pacific from southern Honshu (Japan) to southern China, and coastal Australia from Western Australia to New South Wales (Smith and Böhlke 1997). With more attention to this species, new records have been occurred in different regions, such as Korea (Kim et al. 2012), Vietnam (nearly 12° N, Hibino et al. 2016), the central Philippines (Tañon Strait, Bucol and Alcala 2015, Wagey et al. 2015) and New Zealand (Böhlke and McCosker 2001). Therefore, the present report represents the new identification of the species in Chinese waters and expands its distributional area southward to 5°15'N into South China Sea (Table 3). Meanwhile, Smith and Böhlke (1997) mentioned that the two populations of *G. minor* (the northern and southern populations) are intraspecifically distinguishable by their number of total vertebrae (135–143 in the northern population and 129–135 in the southern population), although there is a slight overlap. The vertebral number of the present specimens agrees well with those of the northern population and do not overlap with the southern population, and similar results are described in other northern populations (Kim et al. 2012, Nakabo 2013, Wagey et al. 2015, Hibino et al. 2016). Hibino et al. (2016) also suggested the two populations of *G. minor* should be regarded as different species. From the NJ tree, two groups were detected among all COI sequences of *G. minor*, of which Group 1 collected from the northern hemisphere matched the northern population and Group 2 collected from Australia matched the southern population. Meanwhile, the genetic distance between both populations has exceeded the threshold of species delimitation. Therefore, we confirm the suggested classification of Hibino et al. (2016). In other case, *Gymnothorax mccoskeri* Smith & Böhlke, 1997 is also an anti-tropical species and distributed in both the northern and the southern hemispheres, but there are no morphological differences between both populations except for a minor variation in head length (Stern and Goren 2013, Hibino et al. 2015). Therefore, related species of *Gymnothorax* with similar distribution show different evolutionary strategy to adapt diverse local habitats, which can drive the formation of new species.

Our study further demonstrate that *G. minor* is distributed in the western Pacific but *G. reticularis* is absent in this region. More specimen collection is necessary in order to define clearly the geographical limits of *G. minor*, especially from Malaysia, Indonesia, or anywhere between 5°15'N and 10° S. Sightings of species differentiation in *G. minor* will be further validated from detailed morphological characteristics and nuclear gene. Specimens of *G. reticularis* are also needed to provide the DNA barcoding based on the correct morphological characteristics.

**Table 3.** Comparative count and measurement characteristics of *G. minor* and *G. reticularis* in different studies.

Source	*G. minor*					*G. reticularis*	
	This study	Hibino et al. (2016)	Kim et al. (2012)	Smith and Böhlke (1997)	Boeseman (1947)	Stern and Goren (2013)	Smith and Böhlke (1997)
Total length (mm)	266.3–552.5	270.5–363.0	469.0	245.0–508.0	450–463.0	460.0	207.0–256.0
**Counts**							
Total vertebrae	137–139	136–139	139	129–143	135–140	123	114–126
Dorsal fin origin	Before of gill opening	Before of gill opening	Before of gill opening	Before of gill opening	Before of gill opening	Before of gill opening	Before of gill opening
Teeth	Serrate	Serrate	Stout serrate	Stout serrate	Stout serrate	Fine serrate	Serrate
Dentition	Uniserial	Uniserial	Uniserial	Uniserial	Uniserial	Uniserial	Uniserial
Median intermaxillary teeth	0–1	0–1	1	0–2	1	1	0
Bars behind gill opening	18–20	19	19	15–22	18–20	18	16–20
**Measurements**							
In % of total length							
Head length	13.5–15.3	12.3–14.6	12.6	10.0–15.2	11.6–11.9	13.1	12.2–15.4
Pre-anal length	45.1–52.1	44.5–44.9	46.7	41.7–47.6	44.8–45.5	46.5	45.5–50.0
Depth at gill-opening	4.5–5.2	4.6–4.7	6.4	4.6–6.3	4.9–5.0	6.4	5.6–6.3
Depth at anus	4.4–5.0	3.6–4.2	5.0	–	–	–	–
Width at gill-opening	2.6–3.9	2.6﻿–3.6	–	–	–	–	–
Width at anus	3.4–3.8	3.2–3.5	–	–	–	–	–
In % of head length							
Snout length	13.4–15.2	13.2–16.4	12.6	12.1–20.0	14.4–15.0	14.4	12.8–17.9
Eye diameter	9.2–10.2	8.9–9.9	10.2	7.7–14.9	7.1–8.9	6.8	10.0–12.8
Interorbital width	12.1–14.8	10.5–13.4	12.1	–	–	–	–
